# A randomized controlled trial to evaluate the effectiveness of a novel mouth rinse in patients with gingivitis

**DOI:** 10.1186/s12903-022-02518-2

**Published:** 2022-11-02

**Authors:** Bianca A. Newman, Claire N. Rosebrough, Ryan A. Tamashiro, Ana P. Dias Ribeiro, Joan A. Whitlock, Gurjit Sidhu, Ikramuddin Aukhil, Dianne Y. Porral, Ann Progulske-Fox, Matthew F. Myntti, Gary P. Wang

**Affiliations:** 1 Department of Periodontology, University of Florida College of Dentistry, Gainesville, FL USA; 2 Division of Infectious Diseases and Global Medicine, Department of Medicine, University of Florida College of Medicine, Gainesville, FL-32610 USA; 3 Department of Restorative Dental Sciences, University of Florida College of Dentistry, Gainesville, FL USA; 4 Next Science LLC, Jacksonville, FL USA; 5 Department of Oral Biology, University of Florida College of Dentistry, Gainesville, FL USA; 6 Infectious Disease Section, Medical Service, North Florida/South Georgia Veterans Health System, Gainesville, FL USA

**Keywords:** Antimicrobial(s), Antiplaque agent(s), Clinical trial(s), Dental hygiene, Gingivitis, Supragingival microbiome

## Abstract

**Background:**

This single-center, randomized controlled trial aimed to determine the effectiveness of a novel, biofilm-disrupting, mouth rinse that combines Cetylpyridinium chloride (CPC) and essential oils in preventing re-accumulation of supragingival plaque and supragingival microbiome in patients with gingivitis after dental prophylaxis.

**Methods:**

One hundred eighteen participants were randomly assigned in a 1:1 ratio to receive twice-daily test mouth rinse (59) or carrier rinse control (59) for 12 weeks after dental prophylaxis.

**Results:**

In a per-protocol analysis that included patients who completed the intervention, the treatment group (39) had significantly lower supragingival plaque scores at 6 and 12 weeks compared to the control group (41; *p* = 0.022). Both groups showed similar improvement in gingivitis score, but neither group had improvement in bleeding score or probing depth. Thirty-eight (29%) patients did not complete the study due to loss of follow-up (17) or early discontinuation of the assigned intervention (21). Microbiome sequencing showed that the treatment rinse significantly depleted abundant and prevalent members of the supragingival plaque microbiome consortium.

**Conclusions:**

Among patients with gingivitis, the novel mouth rinse significantly reduced re-accumulation of supragingival plaque following dental prophylaxis by depleting supragingival plaque microbiome. However, long-term adherence to the rinse may be limited by adverse effects (ClinicalTrials.gov number, NCT03154021).

**Supplementary Information:**

The online version contains supplementary material available at 10.1186/s12903-022-02518-2.

## Background

Chronic periodontitis is a multifactorial disease influenced by smoking, diabetes, and oral hygiene. The initiation of chronic periodontitis is preceded by bacteria in the dental plaque [1] and the imbalanced interaction between subgingival microbiome and the inflammatory response of the host, ultimately leading to tissue destruction and periodontitis [2]. Plaque is a bacterial biofilm that colonizes and adheres to the tooth surface [3], providing protection for the bacteria against mechanical stress as well as antimicrobial agents [4]. Biofilm formation occurs quickly but its maturation requires time. The biofilm initiates an inflammatory reaction through the release of endotoxins as well as through initiation of the host immune response involving regulatory mediators such as transforming growth factor-β1, vascular endothelial growth factor, and transglutaminase 2 [5–7]. Using DNA probes to analyze plaques from both periodontally healthy and diseased patients, Socransky et al. categorized bacterial subgroups on a spectrum from healthy to disease-causing bacteria and defined five major bacterial complexes [8]. Among them, the red complex organisms consisting of *P. gingivalis*, *B. forsythus* (now *T. forsythia*), and *T. denticola* are particularly virulent and have been associated with periodontal breakdown. The presence of red complex bacteria within the sulcus can cause inflammatory reactions and promote the onset of periodontal diseases, presenting initially as gingivitis and then progressing to periodontitis if left untreated.

Non-surgical therapies for the treatment of periodontal disease include scaling and root planning and local delivery of antibiotics, while more invasive surgical options involve resection surgery and regenerative approaches. For these treatment modalities to effectively achieve and maintain periodontal health, they must be combined with adherence to oral hygiene regimens and regular professional maintenance visits. Although these treatment modalities can slow or halt disease progression, the best treatment is prevention. The use of antimicrobial therapies as adjunctive treatments is commonly employed to maintain gingival health or supplement non-surgical or surgical therapies. Toward this goal, Next Science™ has developed an oral mouth rinse that is intended to delay the onset of the periodontal disease process by reducing plaque accumulation and slowing disease progression. The test rinse combines CPC and essential oils, as well as biofilm disrupting technology composed of a high osmolarity solution of sodium hydroxide and potassium phosphate monobasic at pH 9.0. The biofilm disruption effect of this chemistry is achieved by chelation of metal ions in the biofilm extracellular polysaccharide matrix, which cross-link the biofilm together [9–11]. The essential oils in the test rinse include menthol, eucalyptol, methyl salicylate and thymol. Essential oils have antibacterial activity via alteration of the bacterial cell wall [12], and have been shown to reduce plaque and gingival bleeding [13]. Cetylpyridinium chloride is a cationic quaternary ammonium compound with antiseptic and surfactant properties, with antimicrobial effects through the disruption of bacterial cell membranes resulting in alterations of cellular metabolism, and eventual cell death [14]. The test rinse also includes ingredients which chelate metal ions from the biofilm, exposing the bacteria within to the antimicrobial products.

The test oral rinse was previously evaluated in an in vitro study [15], in which its effects against the red complex periodontal pathogens were compared to the effects of Listerine^Ⓡ^ and Perioguard^Ⓡ^. The results showed superior efficacy in reducing the number of red complex bacteria in vitro compared to Listerine, with a greater reduction of both aerobic (supragingival) and anaerobic (subgingival) biofilms compared to Perioguard. Thus, the in vitro data suggest that the test oral rinse may be superior than the current commercially available products in removing or eliminating biofilm and/or preventing accumulation of supragingival plaque. Given its biofilm-disrupting capabilities, the present study aimed to determine the effectiveness of the test oral rinse in reducing re-accumulation of supragingival plaque in patients with gingivitis after dental prophylaxis. In addition, the impact of the test rinse on supragingival microbiome was evaluated.

## Methods

### Investigational product

- Test: Next Science™ OTC Oral Rinse with Essential Oils

(Supplier Sigma Aldrich - Cetylpyridinium chloride monohydrate, meets USP testing specifications, Cinnamaldehyde certified food grade product, Puriss sodium hydroxide, meets analytical specification of Ph. Eur., BP, NF, E524, 98–100.5%, Puriss Potassium phosphate monobasic, anhydrous, ACS reagent, ISO, Ph. Eur., 99.5–100.5%., Menthol, racemic, >.98%, Eucalyptol, Methyl Salicylate, Thymol, Ethanol.)

(Supplier B. Braun - Sterile Water for Irrigation USP)

- Control: OTC Oral Rinse Control

(Supplier Sigma Aldrich – Cinnamaldehyde, certified food grade product)

(Supplier B. Braun – Sterile Water for Irrigation USP)

### Study design and patients

This is a randomized, controlled, double-blind clinical trial at a single center at University of Florida College of Dentistry. Participants were enrolled from the community around the dental school. The inclusion criteria included: age between 18 and 60 years, ability to provide written informed consent, in good general health as determined by the investigator/designee based on a review of the medical history, have at least 20 gradable teeth, and have 10 or more bleeding sites at baseline. The exclusion criteria included: known allergy or sensitivity to components of the oral rinse product including cetylpyridinium chloride (CPC) cinnamaldehyde, sodium hydroxide or potassium dihydrogen phosphate; severe periodontal disease as characterized by purulent exudate, generalized mobility and/or severe recession; current active treatment for periodontal disease, bleeding disorder or use of a blood thinner; current orthodontic treatment; diabetes; pregnancy; antibiotic therapy within 3 months of the baseline visit; or any diseases or conditions that may interfere with the patient safely completing the study as determined by the investigator/designee. All subjects provided written informed consent to participate in the study. This study was approved by University of Florida Institutional Review Board, registered with NIH Clinical Trials Registry on 15/05/2017 (ClinicalTrials.gov NCT03154021).

### Study procedures and treatment

On the morning of each visit, subjects were required to refrain from eating, drinking or performing any oral hygiene efforts. Visits were conducted in the morning to encourage compliance. At screening, baseline measurements for gingival index as proposed by Loe (1967) [16], the Turesky modification of the Quigley and Hein Plaque index [17], and probing depths were determined. Plaque samples were collected from the following four sites: mesial surface of maxillary first molar on the left side, distal surface of maxillary first premolar on the right side, mesial surface of mandibular second molar on the left side, and distal surface of mandibular lateral incisor on the right side. At each visit, all four plaque samples were collected from the supragingival tooth surface with a sterile curette and pooled in a single tube. If any of these four teeth were missing, the next adjacent tooth was used to collect the plaque sample. All plaque samples were placed immediately on ice and stored at − 80 °C until further processing.

At the second visit (within 2 weeks of the baseline visit), a professional prophylaxis was completed by a single hygienist, and then participants were randomized 1:1 to receive either treatment mouth rinse or control carrier rinse. A randomization schedule was provided by the sponsor, and the treatment and placebo products were coded numerically so that the study site staff and the subjects were blinded to the identity of the products. Patients were given standardized toothbrushes and toothpaste and were instructed not to use any additional oral hygiene aids. Flossing, toothpicks, interdental brushes or use of additional mouth rinses was not permitted during the 12-week study. Rinsing was performed twice daily with 20 mL of the assigned mouth rinse. Patients were instructed to rinse for 30 seconds and expectorate. Clinical measurements were repeated following 6 and 12 weeks of mouth rinse use, and supragingival plaque samples were collected from the same four sites at 12 weeks. The primary outcome was improvement in plaque index at 6 and 12 weeks. The secondary outcomes were changes in gingival index, bleeding score, pocket depths, and changes in the composition of supragingival microbiome. Safety assessments included the type, incidence, and severity of adverse events that occurred during the study period.

### Statistical analysis

The planned sample size was estimated to provide 80% power (one-sided, at a 5% significance level), assuming an effect size (treatment difference in plaque index divided by within subject standard deviation) of at least 0.528, to detect a difference between the Test Mouth Rinse and the Carrier Control. Plaque score, gingivitis score, bleeding score, and probing depths of subjects were analyzed using the General Linear Model followed by Tukey’s post-hoc test, and a *p*-value of less than 0.05 was considered statistically significant.

### Supragingival microbiome assessment

Supragingival microbiome was analyzed using 16S rRNA sequencing on the Illumina platform. Detailed procedures for sequencing and bioinformatics analysis are described in Supplementary Methods.

## Results

### Patient characteristics

**Fig. 1:**
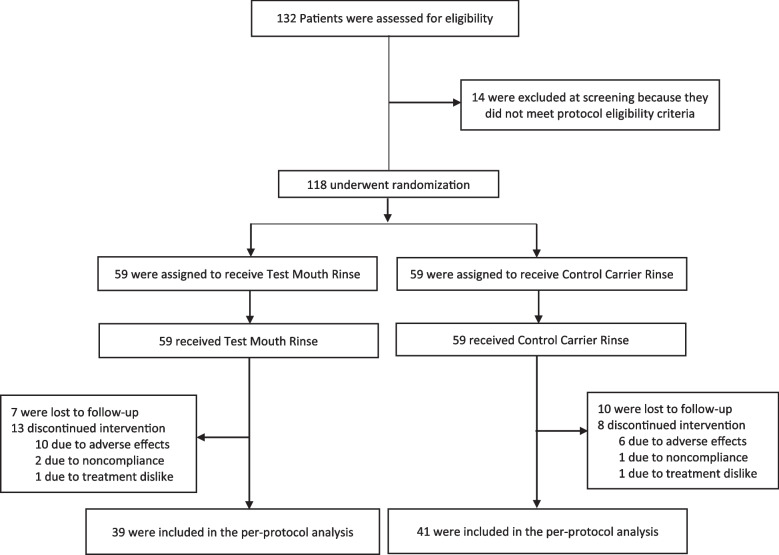
Randomization and follow up of the patients

From July 2017 and May 2018, a total of 132 patients were screened in dental clinics at the University of Florida College of Dentistry. Of these, 14 did not meet the protocol eligibility criteria, and 118 adult patients underwent randomization (Fig. 1). Seventeen of 118 participants (14%) were lost to follow-up, and 21 participants (18%) discontinued intervention during the study. Overall, 80 participants (39 subjects in the test mouth rinse group and 41 in the carrier control group) completed the study. Baseline characteristics of the participants in the trial groups were comparable (Table 1).

**Table 1:** Baseline Characteristics of the Participants

Characteristic	Test Mouth Rinse (***N*** = 39)	Carrier Control Rinse (***N*** = 41)
Median age (range) - years	25 (18—55)	28 (19—56)
Sex		
Male sex	12 (31%)	17 (41%)
Female sex	27 (69%)	24 (59%)
Race		
White	30 (77%)	30 (73%)
Black or African American	2 (5%)	2 (5%)
Asian	7 (18%)	6 (15%)
Native Hawaiian or Other Pacific Islander	0	1 (2%)
Other	0	2 (5%)
Ethnicity		
Hispanic or Latino	10 (26%)	4 (10%)
Not Hispanic or Latino	29 (74%)	37 (90%)
Median height (range) – in.	65 (58—73)	67 (62—74)
Median weight (range) – lb.	154 (95—235)	168 (110—255)
Baseline Clinical Measures		
Gingivitis Score	1 ± 0.29	1.06 ± 0.37
Bleeding Score	0.99 ± 0.62	1.1 ± 0.72
Pocket Depth (mm)	2.5 ± 0.8	2.5 ± 0.8
Plaque Score	2.61 ± 0.45	2.58 ± 0.43

### Efficacy

Plaque score was significantly affected by treatment group (*p* = 0.022), with a significant difference between test rinse and control; time was not a significant variable (*p* = 0.51) (Table 2). Gingivitis score was significantly affected by time (*p* = 0.014), with a significant reduction at week 12 vs. either of the prior two time points; treatment group was not a significant variable (*p* = 0.34). In contrast, neither bleeding score nor probing depths changed significantly over time in either group, and no significant difference was observed between the two groups. These results showed that the test rinse significantly reduced re-accumulation of supragingival plaque following dental prophylaxis compared to the carrier control rinse. However, its effects on gingival inflammation was similar to the carrier control.

**Table 2:** Gingivitis score, bleeding score, probing depths, and plaque score of subjects randomized to the test mouth rinse or carrier control rinse at baseline, 6 weeks, and 12 weeks. For each of the four clinical measurements, data between the test and control rinse and between time points were compared using General Linear Model followed by Tukey’s post-hoc test. For statistical differences, see text

Time Points (weeks)	Gingivitis Score		Bleeding Score	Probing Depth			Plaque Score	
	Control	Test	Control	Test	Control	Test	Control	Test
0	1.06 ± 0.37	1 ± 0.29	1.1 ± 0.72	0.99 ± 0.62	2.5 ± 0.8	2.5 ± 0.8	2.58 ± 0.43	2.61 ± 0.45
6	0.97 ± 0.41	1.02 ± 0.26	0.81 ± 0.71	0.81 ± 0.61	2.4 ± 0.8	2.4 ± 0.7	2.55 ± 0.43	2.32 ± 0.38
12	0.85 ± 0.37	0.87 ± 0.42	0.76 ± 0.58	0.71 ± 0.51	2.3 ± 0.8	2.3 ± 0.8	2.61 ± 0.38	2.34 ± 0.4

### Safety

Adverse events were reported in 22 of 59 patients (37%) who received the test rinse and in 11 of 59 patients (19%) who received placebo during the study period (Table 3). Sixteen subjects (Test rinse: 10 [17%]; control: 6 [10%]) discontinued the study due to the adverse effects (AE). The most common adverse events in the test rinse group were oral lesions (in 8 patients [14%], Supplementary Fig. 1), teeth staining (in 6 patients [10%]), white film or plaque (in 6 patients [10%]), gingival sloughing (in 4 patients [7%]), loss or alteration of taste (in 2 patients [3%]), and tongue numbness (in 1 patient [2%]). The incidence of oral irritation was higher in the placebo group (in 8 patients [14%]) than those in the test rinse group (in 5 patients [8%]). Adverse events that were moderate in severity occurred in 2 patients [3%] in the test rinse group (erosion of mucosa and tissue irritation) and in 2 patients [3%] in the placebo group (tissue irritation). Severe adverse events occurred in 2 patients [3%] in the placebo group (inflamed papilla with heavy bleeding on probing (BOP) and gallbladder surgery). The latter AE was determined to be a serious adverse event. The remaining AEs were considered mild. All adverse events resolved spontaneously, or with intervention (such as teeth cleaning for brown stains), or with the cessation of the assigned study product.

**Table 3:** Adverse Events

	Test Mouth Rinse (*n* = 59)	Carrier Control Rinse (n = 59)
Any adverse events	22 (37%)	11 (19%)
Adverse events that were considered moderate in severity	2 (3%)	2 (3%)
Adverse events leading to discontinuation of test rinse or placebo	10 (17%)	6 (10%)
Most common adverse events		
All events	29 (49%)	14 (24%)
Oral Lesions	8 (14%)	3 (5%)
Staining	6 (10%)	0
Loss/Alteration of Taste	2 (3%)	0
Oral Irritation: Erythema/Burning/Inflammation	5 (8%)	8 (14%)
White film/plaque	6 (10%)	0
Tongue Numbness	1 (2%)	0
Gingival Sloughing	4 (7%)	1 (2%)
Other	0	1 (2%)
Total	29	14

### Microbiome assessment

**Fig. 2:**
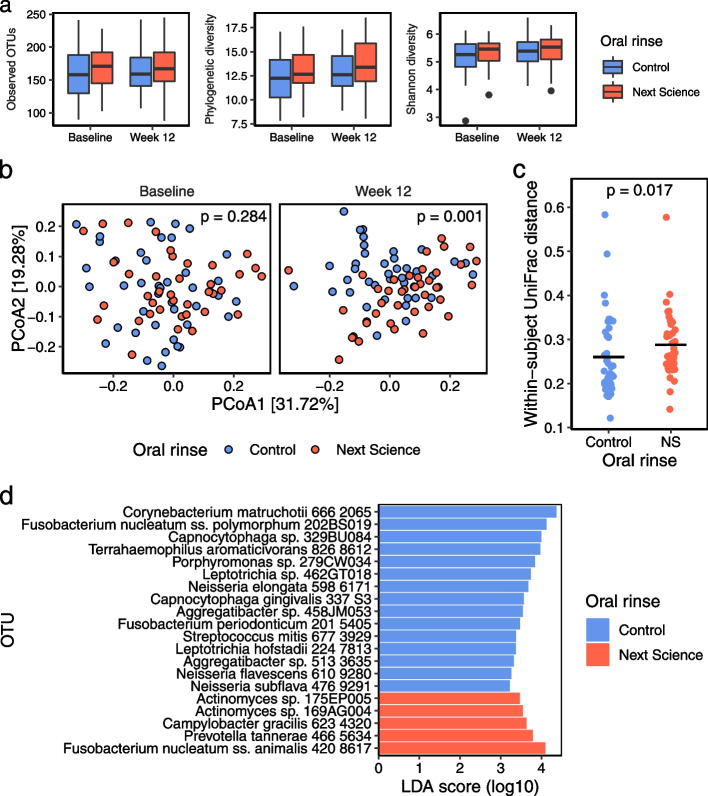
Shifts in the supragingival microbiome after 12 weeks of oral rinse use. **a** Three alpha diversity metrics comparing oral rinses over time. Species richness was estimated by the number of observed OTUs and Faith’s phylogenetic diversity, and species diversity was measured by Shannon’s diversity index. Boxplots show the median and interquartile range (IQR). Whiskers extend up to 1.5 x IQR with points beyond representing outliers. See Supplementary Table for significance tests. **b** Principal coordinates analysis on weighted UniFrac distances comparing the treatment and the control groups at baseline and at 12 weeks. *P* values were generated using permutational multivariate analysis of variance with 999 permutations. **c** Comparison of the longitudinal change in the microbiome over 12 weeks of rinse use. Within-subject UniFrac distance quantifies the change in a single individual’s microbiome over time, with greater distances representing more change. Black bars show the mean within-subject UniFrac distance, and *p* value was generated using a Wilcoxon rank sum test. **d** Top 20 taxa that were most differentially abundant between the two groups after 12 weeks of oral rinse use. Linear discriminant analysis with effect size identified specific OTUs differentially enriched in the control or the treatment group, indicated by color. See Supplementary fig. 2 for all differentially abundant OTUs. NS: Next Science oral rinse, OTU: Operational taxonomic unit

16s rRNA sequencing analysis of the supragingival microbiome identified 538 unique operational taxonomy units (OTUs) belonging to 10 different phyla. We first compared the temporal trends in species richness and diversity between those who received the test mouth rinse and the carrier control rinse. At baseline, samples from the control and the test rinse treatment subjects had similar levels of richness and diversity (Fig. 2a, Supplementary Table). At 12 weeks, species richness and diversity increased in both groups, but the differences were not statistically significant. We then examined the effects of the test rinse on the overall community structure compared to the control rinse. Differences in community structure was measured using weighted UniFrac distance metric, a distance metric that accounts for phylogenetic relatedness and abundance of OTUs in each sample. The communities of control and treatment groups were similar at baseline (Fig. 2b; PERMANOVA: F = 1.23, *p* = 0.284), but diverged over time. At 12 weeks, samples cluster according to their experimental group (Fig. 2b; PERMANOVA: F = 4.87, *p* = 0.001). Indeed, differences between baseline and week 12 samples from the same subject were 18% greater in the treatment group than the control group (Fig. 2c, Wilcoxon rank sum: W = 552, *p* = 0.017).

Using LEfSe, we identified 33 OTUs that were overabundant in the control group and 40 OTUs that were overrepresented in the treatment group at week 12 (Supplementary Fig. 2). The OTUs that were differentially depleted in the treatment group (i.e. enriched in the control group) included *Corynebacterium matruchotii*, *Corynebacterium durum, several Actinomyces, Fusobacterium, Leptotrichia, Capnocytophaga, Neisseria, Streptococcus, Aggregatibacter, Porphyromonas, Terrahaemophilus aromaticivorans,* and *Lautropia*, all of which are abundant and prevalent members of the supragingival plaque microbiome [18]. Thus, the microbiome analysis demonstrated that the test rinse significantly reduced re-accumulation of dominant members of the supragingival plaque microbiome. Top 20 OTUs that were most differentially abundant between the two groups are shown in Fig. 2d.

## Discussion

Several studies have reported the effects of a Cetylpyridinium chloride (CPC) rinse vs. placebo rinse, an essential oil rinse vs. placebo rinse, and a CPC rinse vs. an essential oils rinse [13, 14, 19, 20]. However, to our knowledge, this is the first study that evaluates the clinical and microbiological effects of a mouth rinse that combines CPC and essential oils with a biofilm disrupting formulation. Our results showed that in gingivitis patients, the test rinse significantly reduced re-accumulation of supragingival plaque by depleting the abundant and prevalent members of the supragingival plaque microbiome following dental prophylaxis. However, adverse effects may limit long-term adherence to the rinse.

Our study examined the impacts on the supragingival plaques. There were several reasons for focusing on supragingival plaques. First, gingivitis patients harbor less complex subgingival periodontal biofilm with fewer red complex bacteria, and this study included only patients with gingivitis and excluded patients with periodontal disease. Second, Wunderlich showed that mouth rinses only penetrate an average of 0.2 mm below the gingival margin [21]. If at home use of the mouth rinse is not physically reaching the subgingival environment, it is reasonable to focus on the supragingival plaque where the rinse is in direct contact. Third, even in a clinical setting, irrigation of a pocket (PD ≥ 5 mm) with an irrigating syringe penetrates an average of 1.8 mm subgingivally [22]. When looking at other oral hygiene aids, toothbrushes have been shown to penetrate an average of 0.9 mm subgingivally [23]. Waterpik® at a high-pressure setting may deliver solution to an average of halfway between the free gingival margin and the most coronal connective tissue attachment [24]. These may be an adequate depth for our gingivitis patients, but our study was testing the results of at-home use of the mouth rinse without any adjunctive oral hygiene aids. Thus, future studies on the effects of the test rinse in combination with irrigating syringes or Waterpik® devices may be warranted.

Our results showed that following dental prophylaxis, the test rinse significantly reduced re-accumulation of supragingival plaque with effects sustaining through 12 weeks. The gingival index score also improved but the difference between the two groups was not statistically significant. The reason for similar improvements in gingivitis score between the two groups may be attributed to the prophylaxis that each patient received after the baseline visit, as the benefits of dental prophylaxis for improvement in gingivitis are widely accepted. The frequency of dental visits prior to this study was widely variable and many patients had not had professional cleanings in recent years. Furthermore, Hawthorn effect could have been a contributing factor—home oral hygiene may have improved because the subjects were aware that they were participating in a dental study and knew that gingivitis and plaque scores were being evaluated.

The supragingival plaque communities have been shown recently to have an organized spatial structure of a consortium consisting of aerobes and facultative anaerobes in a distinctive community called “hedgehog” based on combinatorial labeling and spectral imaging FISH analysis [18]. In this hedgehog structure, filaments of *Corynebacterium* spp. (primarily *C. matruchotii and C. durum* in most individuals) are densely packed at the base serving as the foundation or a nucleating species to bind to a biofilm on the tooth surface containing *Streptococcus* and *Actinomyces*. These *Corynebacterium* filaments extend out through an annulus in the middle layer that is occupied by elongated filaments of *Fusobacterium*, *Leptotrichia*, and *Capnocytophaga*, and the distal tips of the *Corynebacterium* filaments are surrounded by cocci including *Streptococcus* and *Porphyromonas,* as well as *Haemophilus*/*Aggregatibacter* and *Neisseriaceae* to form “cornbob” structures. Interestingly, our microbiome analysis showed that nearly all microbial taxa that were differentially depleted in the treatment group were also organisms previously identified in the hedgehog structure (Fig. 2d and Supplementary Fig. 2). Thus, our microbiome data demonstrate that the test rinse reduced the re-accumulation of supragingival plaques by depleting members of the hedgehog consortium in the plaque microbiome. Moreover, Unifrac analysis showed that the supragingival plaque microbiome in the treatment group shifted substantially compared to the controls (Fig. 2b), with the two groups diverging significantly at 12 weeks. We note that several microbial taxa associated with gingivitis and periodontitis, including *Fusobacterium nucleatum, Tannerella forsythia,* and *Treponema socranskii,* were enriched in the treatment group. The significance of this observation is unclear, but suppression of dominant members of the hedgehog consortium by the test rinse may have led to the over-representation of previously low abundant organisms in the plaque microbiome. It would be important in future studies to determine whether enrichment of these putative periodontal pathogens and the microbiome shifts induced by the test rinse are beneficial for the maintenance of oral health, or are harmful for progression toward disease. Furthermore, as several biomarkers (e.g. TGF-β1, transglutaminase 2, NLRP3) have been implicated in the pathogenesis of periodontitis [5–7], it would be of interest to evaluate the effects of the test oral rinse on these early biomarkers.

The study had several limitations. First, a significant proportion of patients (10/59 or 17% in the treatment group and 6/59 or 10% in the placebo group) discontinued the study due to adverse effects. This is a potential drawback that may limit long-term adherence to the oral rinse. Second, the study was conducted in participants with gingivitis, and thus its effect on preventing gingivitis in patients without gingivitis and changing the microbiome of their supragingival plaque could not be ascertained. Third, the study follow-up was limited to 12 weeks, and thus it was not possible to determine any long-term preventive or therapeutic effects of the test rinse.

## Conclusions

A rinse combining CPC and essential oils with a biofilm disrupting chemistry reduces re-accumulation of supragingival plaque and the associated supragingival microbiome following dental prophylaxis. However, the test rinse did not improve gingivitis score significantly compared to carrier controls. Future research should evaluate the long-term effects of the test rinse in reducing or preventing gingivitis, and determine whether the combination of these ingredients is more effective than either the CPC rinse or essential oil rinse alone and how each component impacts the supragingival microbiome.

## Supplementary Information

**Additional file 1.**

## Data Availability

The data that support the findings of this study are openly available in DANS (Data Archiving and Networking Services) at 10.17026/dans-xne-pwfn [DOI(10.17026/dans-xne-pwfn)].

## Abbreviations

CPC: Cetylpyridinium chloride
AE: Adverse effects
BOP: Bleeding on probing
OTUs: Operational taxonomy units
