# Supplementary material for: A randomized controlled trial to evaluate the effectiveness of a novel mouth rinse in patients with gingivitis
Source: BMC Oral Health. 2022 Nov 2;22:461. doi: 10.1186/s12903-022-02518-2 (PMC9632080; doi:10.1186/s12903-022-02518-2)
Supplement: Supplementary file 1 — Additional file 1. [file 12903_2022_2518_MOESM1_ESM.docx]

**Supplementary Methods**

**Microbiome Assessment**

Bacterial DNA was extracted from baseline and week 12 supragingival plaque samples using the DNeasy Powersoil Kit (Qiagen, Venlo, Netherlands) per the manufacturer’s protocol with the following modifications. An SDS-containing solution was added to PowerBead tubes to facilitate cellular lysis and membrane breakdown. Tubes were secured to a Vortex Adapter, mixed for ten minutes, and heated at 70°C for 10 minutes. The supernatant was transferred to a clean, 2 mL collection tube and a solution which precipitates non-DNA material was added. Samples were incubated at 4°C for 10 minutes, centrifuged, and the supernatant was transferred to another clean tube. This step was repeated, and then a salt solution was added to facilitate DNA binding to the spin column. Samples were loaded onto spin columns and washed twice to remove contaminants. Purified DNA was eluted from the column and stored at -20°C until further processing.

The 16S rRNA gene (V1-V3 region) was amplified using primers 27F (‘5-AGAGTTTGATCCTGGCTCAG-3’) and 534R (5’-ATTACCGCGGCTGCTGG-3’) with barcodes to allow multiplex deep sequencing as described previously (Kozlov et al. 2018). The final PCR reactions contained 0.75 U Accuprime Taq High Fidelity Polymerase (Invitrogen, Carlsbad, CA), 2 μL 10X PCR buffer II, 100 nM forward primer, 100 nM reverse primer, 2 μL purified DNA template, and DNA/RNA-free water in a total reaction volume of 20 μL. Cycling conditions were as follows: denaturation at 95°C for 2 minutes, 25 cycles of denaturation at 95°C for 20 seconds, annealing at 56°C for 30 seconds and extension at 72°C for 5 minutes. PCR products were analyzed on a SYBR Safe 1% agarose gel (Invitrogen, Carlsbad, CA) to ensure an expected size of 600 base pairs. The amplicons were excised, purified with NucleoSpin Gel and PCR Clean-up kit (Macherey-Nagel, Bethehem, PA), and then quantified with Qubit HS DNA quantification kits (Invitrogen, Carlsbad, CA). Equimolar concentrations of DNA were then pooled and purified. qPCR was performed on the pool using the Library Quant Kit (Kapa Biosystems, Wilmington, MA). 16S rRNA sequencing was performed on the Illumina MiSeq platform using MiSeq Reagent kit V3 and PhiX control V3 kit (Illumina, San Diego, CA).

Raw MiSeq paired-end reads of 300 nucleotides each (covering the V1–V3 hypervariable region of the 16S rRNA gene using primers 27F and 534R) were processed using custom scripts in R (R Core Team 2018). The reads were filtered based on matches to barcode/primer and an average quality score of 30. Samples were de-multiplexed according to their unique combination of barcodes (4 to 8 nucleotides) on each paired end. The barcodes and primers were trimmed for downstream analysis. To reconstruct the original amplicon, paired-end reads were joined using FLASh (Fast Length Adjustment of Short reads), with a minimum overlap of 10 base pairs. USEARCH alignment was employed with a 97% identity and 80% aligned query threshold to assign taxonomic information from the Human Oral Microbiome Database (Chen et al. 2010) to each read. Reads that did not meet filtering criteria were excluded from additional analysis.

The OTU (operational taxonomic unit) table was subsampled down to an even sampling depth of 4300 reads per sample for alpha and beta diversity analysis using the core-metrics-phylogenetic pipeline in QIIME2 (version 2018.8, <https://qiime2.org/>). Alpha diversity was estimated using two species richness metrics (observed OTUs and Faith’s phylogenetic diversity) and one species diversity metric (Shannon’s diversity). The weighted UniFrac distance metric measured the beta diversity, which quantifies the differences between two communities.

For microbiome analysis, statistical analysis were performed in R 3.4.2 (R Core Team 2018) unless otherwise noted. Mixed linear models using the lmer() in lme4 v.1.1.19 (Bates et al. 2015) tested for differences in alpha diversity over time with respect to each treatment. Week was treated as a nominal variable, and subject identity was included as a random effect. Clustering of samples was visualized with principal coordinates analysis (PCoA) using the betadisper() in vegan v.2.5-3 (Oksanen et al. 2012). Significance of these clusters was tested with Permutational multivariate analysis of variance (PERMANOVA) using the adonis() in vegan v.2.5-3 (Oksanen et al. 2012). The treatment and control groups were compared at baseline and again 12 weeks later. Change in the microbiome over time was measured as the within-subject UniFrac distance, and treatment and control groups were compared using the Wilcoxon rank-sum test. Differential abundance analysis was conducted on the unrarefied OTU table using LEfSe (Linear Discriminant Analysis with Effect Size) (Segata et al. 2011) on Galaxy 1.0. OTUs were considered differentially abundant if the LDA score > 2.0.

**References**

Bates D, Mächler M, Bolker BM, Walker SC. 2015. Fitting linear mixed-effects models using lme4. J Stat Softw. 67(1):1-48.

Chen T, Yu WH, Izard J, Baranova OV, Lakshmanan A, Dewhirst FE. 2010. The Human Oral Microbiome Database: a web accessible resource for investigating oral microbe taxonomic and genomic information. The Forsyth Institute, Oxford. [updated 2010; accessed 26 April 2022]; Vol. 2010. https://www.homd.org/.

Kozlov A, Bean L, Hill EV, Zhao L, Li E, Wang GP. 2018. Molecular identification of bacteria in intra-abdominal abscesses using deep sequencing. Open Forum Infect Dis. 5(2).

Oksanen AJ, Blanchet FG, Kindt R, Legen- P, Minchin PR, Hara RBO, Simpson GL, Solymos P, Stevens MHH. 2012. Vegan: Community ecology package. [updated 2012; accessed 26 April 2022]. <http://mirror.bjtu.edu.cn/cran/web/packages/vegan/vegan.pdf/>.

Segata N, Izard J, Waldron L, Gevers D, Miropolsky L, Garrett WS, Huttenhower C. 2011. Metagenomic biomarker discovery and explanation. Genome Biol. 12(6).

**Supplementary Figures**


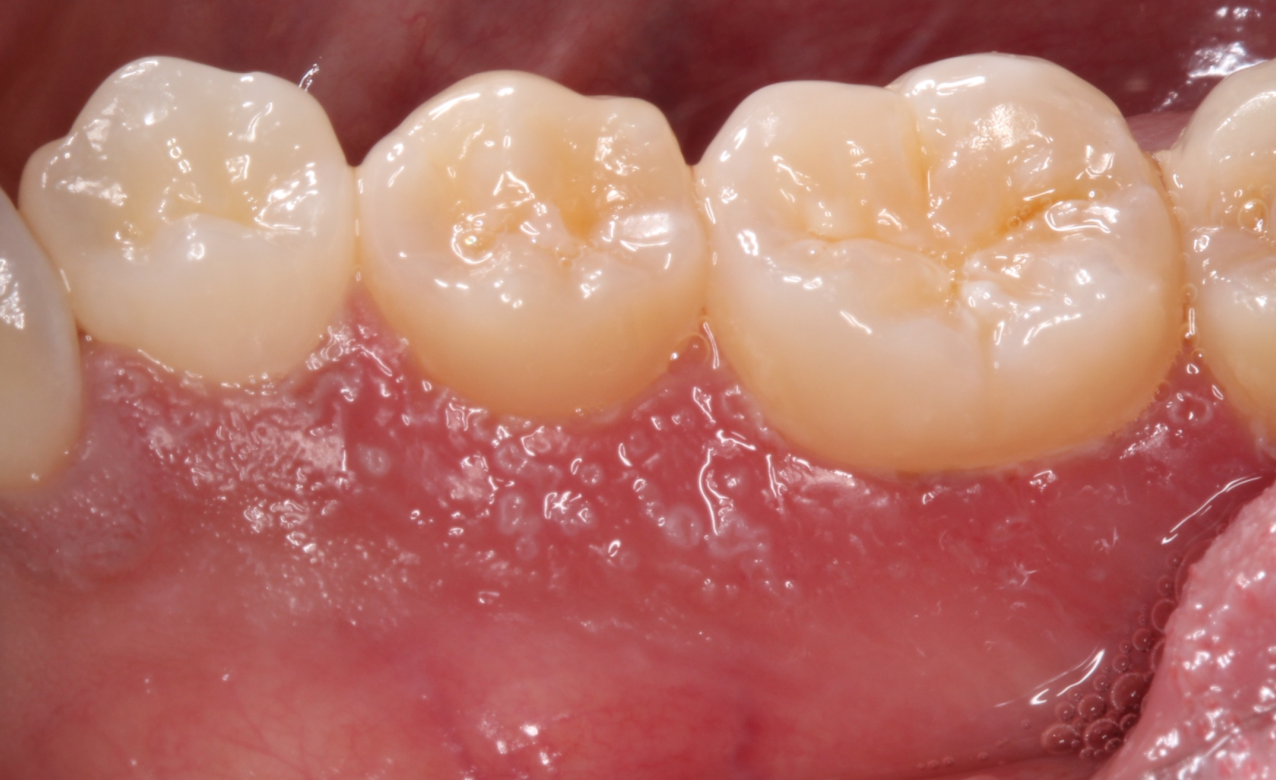


**Supplementary Figure 1.** Small, white round granules on the gingival and mucosal regions in some subjects who discontinued the study.


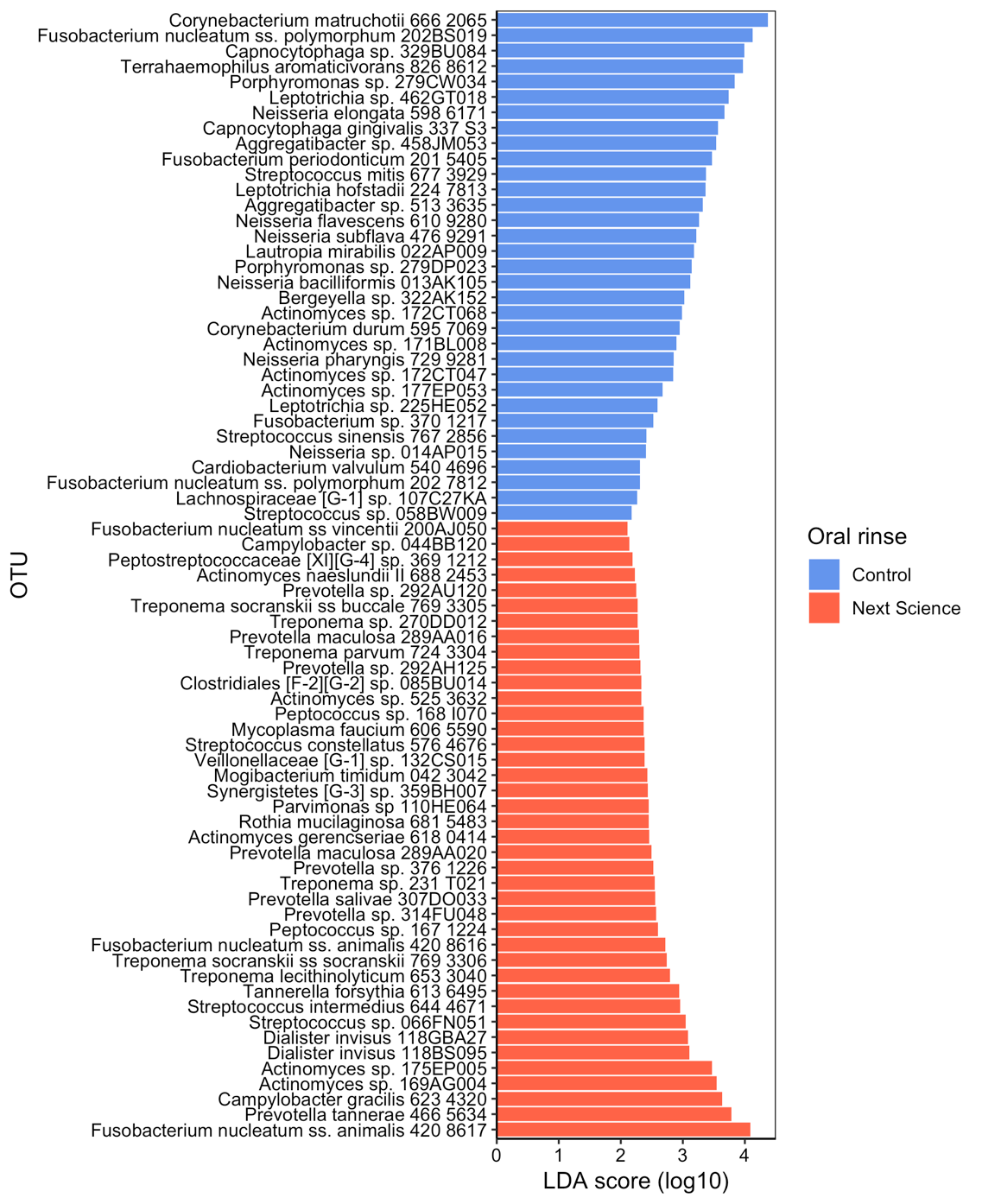


**Supplementary Figure 2**. Differentially abundant OTUs determined by linear discriminant analysis with effect size comparing the two groups at 12 weeks. Color indicates enrichment of an OTU in each group.

**Supplementary Table:** Mixed linear models of species richness and diversity. Mixed linear models were used to determine the interaction between mouth rinse treatment and time. Model coefficients are shown with p-values in parenthesis. Reference groups for the linear mixed models are shown next to the predictors.

|  | Diversity Metric | | | | |
| --- | --- | --- | --- | --- | --- |
| Model Predictors | Observed OTUs |  | Faith’s phylogenetic diversity |  | Shannon’s diversity |
| Treatment  (ref: control) | 9.163 (0.243) |  | 0.728 (0.158) |  | 0.109 (0.387) |
| Time  (ref: baseline) | 3.585 (0.430) |  | 0.543 (0.054) |  | 0.141 (0.176) |
| Treatment x Time | -4.867 (0.454) |  | -0.141 (0.724) |  | -0.055 (0.710) |
